# There is no such thing as interoception

**DOI:** 10.3389/fpsyg.2025.1488415

**Published:** 2025-02-10

**Authors:** Felix A. Schoeller, Ben Zhang, Teresa Garcia, Nicco Reggente

**Affiliations:** Institute for Advanced Consciousness Studies, Santa Monica, CA, United States

**Keywords:** interoception, measures, methods, cardioception, respiroception, gastroception, interoceptive accuracy, interoceptive sensitivity

## 1 Introduction

Interoception has emerged as a prominent construct in psychology, neuroscience, and medicine, referring to the perception and processing of signals originating from within the body (Craig, 2002; Khalsa et al., 2018). It is thought to play a crucial role in emotional experience, self-regulation, and various clinical conditions (Barrett and Simmons, 2015; Tsakiris and Critchley, 2016). The notion of interoception as a coherent system spanning different bodily domains has gained traction, with researchers typically treating it as a unitary ability. However, mounting evidence challenges this conceptualization. In fact, large variability exists in the accuracy of different interoceptive channels (Vaitl, 1996; Ferentzi et al., 2017, 2018; Harver et al., 1993; Whitehead and Drescher, 1980; Garfinkel et al., 2016, 2017).

While the title of this article is intentionally provocative, it serves to highlight a critical issue in the field: namely that the term “interoception” is often used in ways that belie the complexity and diversity of the phenomena it purports to describe. The evidence provided herein focuses primarily on accuracy across different *modalities*, though we acknowledge that interoception encompasses multiple dimensions beyond accuracy (e.g., sensibility, awareness, attention, intensity). Even within this focused scope, the data strongly suggests that treating interoception as a unitary construct is problematic. Such a monolithic view of interoception mirrors both the tension between categories (i.e., classes of entities grouped by shared features) and concepts (i.e., mental representations capturing a category's essence), reminiscent of the perception of a mosaic: from afar, interoception appears as a cohesive category or single image, but closer inspection reveals it as a complex concept comprising distinct, often unrelated subconcepts—akin to individual tiles.

In fact, studies consistently find weak or absent correlations in behavioral performance across interoceptive tasks probing different modalities, such as cardiac, respiratory, and gastrointestinal perception (Ferentzi et al., 2018; Garfinkel et al., 2016; Harver et al., 1993). Associations between interoceptive indices and affective and clinical variables also vary across domains (Baranauskas et al., 2017; Paulus and Stein, 2010). In the next sections, we review evidence for the dissociability of interoceptive abilities across modalities. We focus on the more extensively studied perceptions: cardiac, respiratory, and thermal signals.^1^ Our central thesis is that progress or training in one interoceptive domain does not necessarily translate to others, contrary to the notion of a unitary interoceptive ability.

This dissociability is akin to athleticism in sports: while proficiency in tennis might transfer to pickleball due to similar demands, there's minimal skill transfer between disparate sports like golf and water polo, which require vastly different physical aptitudes. We argue that interoception, like athleticism, is best understood as a useful heuristic for organizing distinct lines of research, rather than a coherent empirical phenomenon. This perspective has important implications for the study and treatment of disorders thought to involve aberrant interoception. Deficits in one modality may not imply deficits in others, *necessitating the development of a comprehensive multi-modal assessment*, much like how athletic potential in one sport doesn't guarantee success across all sports. Interventions targeting specific interoceptive domains may be more effective than general interoceptive training, just as sport-specific training often yields better results than general fitness regimens. By embracing a differentiated view of interoception, we can develop more precise and valid models of its role in health and disease, acknowledging the unique demands and characteristics of each interoceptive modality.

## 2 The case for dissociable interoceptive abilities

### 2.1 The problem with interoceptive tasks

Interoceptive accuracy is typically assessed through various modality-specific tasks, each designed to measure sensitivity to different internal bodily signals. However, these tasks face several methodological challenges that can impact their validity and reliability. The Heartbeat Counting Task (HCT) is the most widely used measure of cardioceptive accuracy (and is often mischaracterized as a measure of “interoception”), but its validity has been questioned. Studies have shown that HCT performance is influenced by non-interoceptive processes, including time estimation and prior knowledge of heart rate (Desmedt et al., 2018, 2020; Ring and Brener, 1996). Intelligence has been found to mediate HCT performance through knowledge of resting heart rate (Murphy et al., 2018). Meta-analyses have revealed weak or non-significant associations between HCT performance and mental health outcomes (Desmedt et al., 2022). Respiratory and gastric perception are much less studied, however their instruments have similar issues. Respiroceptive accuracy is typically assessed with the Respiratory Resistance Sensitivity Task (RRST), where participants judge breathing difficulty under varying resistances (Nikolova et al., 2022). RRST can be influenced by factors such as anxiety and asthma (Ritz et al., 2000), limiting its applicability in certain populations. The Water Load Test (WLT) measures gastric interoception by having participants drink water to perceived fullness (van Dyck et al., 2016). However, this test can be affected by factors like recent food intake, hydration status, and individual differences in stomach capacity (Mejía-Rivas et al., 2009). Thermoceptive accuracy, assessed via a classic temperature task or dynamic thermal matching task, has been shown to vary based on the amount of hair on the skin of participants (Crucianelli et al., 2021, 2022).

Given these limitations, considerable efforts have been made by researchers to develop alternative tasks to more accurately assess interoceptive accuracy (review in Garfinkel et al., 2022; Schoeller et al., 2024; Weng et al., 2021; Desmedt et al., 2023). For instance, the Heartbeat Discrimination Task (HDT) minimizes reliance on knowledge of heart rate and time estimation (Hickman et al., 2020; Brener and Kluvitse, 1988). Signal detection theory has also been applied to interoceptive tasks to separate sensitivity from response biases (Garfinkel et al., 2015, see also Ring et al., 2015; Larsson et al., 2021). Furthermore, tasks like the Respiratory Occlusion Detection Task and the use of gastric balloon distension offer more precise measures for respiratory and gastrointestinal interoception,^2^ respectively (Faull et al., 2017; Herbert et al., 2012). Researchers have also turned to examine the relationships between different interoceptive channels and the potential for more comprehensive, multimodal assessments of interoception.

### 2.2 Weak correlations across interoceptive tasks

A key line of evidence for the dissociability of interoceptive modalities comes from studies examining correlations in performance across tasks (Schoeller et al., 2024). If interoception reflects a unitary ability, one would expect individuals who excel in one domain to perform well in others. However, this is rarely the case. Garfinkel et al. (2016) assessed interoceptive accuracy in cardiac and respiratory domains using heartbeat discrimination and respiratory resistance tasks. They found no significant correlation between accuracy scores on the two tasks, suggesting distinct underlying abilities. Similar findings have emerged in other studies comparing cardiac and respiratory perception (Ehlers et al., 2000; Pollatos et al., 2005). The lack of concordance extends beyond cardiac and respiratory perception. Ferentzi et al. (2018) administered a battery of tasks spanning cardiac, respiratory, gastrointestinal, and pain perception domains to a large sample. Correlations between accuracy scores were weak and mostly non-significant, indicating little shared variance. In another study, Crucianelli et al. (2022) found that no relationship was found between thermoceptive and cardioceptive accuracy. Similar patterns were found when comparing interoceptive abilities across somatic and visceral modalities (Michael et al., 2015; Steptoe and Vögele, 1992). Even within a given interoceptive domain, different task variants often yield discrepant results. For example, heartbeat counting and discrimination tasks frequently show low or absent correlations (Ring and Brener, 1996). Evidence also suggests cardioceptive accuracy differs between males and females (Fairclough and Goodwin, 2007). This suggests that even ostensibly similar tasks may tap into distinct facets of interoception. Alternatively, it is possible that the apparent lack of convergence among different interoceptive tasks reflects limitations in the tasks themselves, and the need for more rigorously validated and sensitive measures, that accurately capture underlying computations. Indeed, studies with interventional methodologies that actively modulate physiological states—such as controlled water loading to satiation—have found that cardiac awareness is related to greater sensitivity for gastric functions, suggesting that there is a general sensitivity for interoceptive processes across the gastric and cardiac modality (see Herbert et al., 2012).

The weak correlations across interoceptive modalities are striking given the shared neuroanatomical substrates thought to underlie interoception, such as the insula and anterior cingulate cortex (Craig, 2009; Critchley et al., 2004). While these regions integrate signals from different bodily systems, the behavioral evidence suggests that this integration does not give rise to a unitary ability. Indeed, this is an oversimplification. The insula is a widely heterogeneous structure supporting distinct interoceptive modalities via several divergent neural pathways. Engelen and Solcà (2023) showed how fundamental bodily rhythms—particularly cardiac, respiratory, and gastric cycles—entrain and shape neural activity throughout the brain. Their work highlights how mechanoreceptors relay visceral signals via vagal and spinal pathways to integrative hubs like the insula and anterior cingulate cortex. These inputs, in turn, modulate widespread cortical and subcortical networks, influencing perception, cognition, and emotion. The same mismatch between conceptualization and measurement is reviewed by Desmedt and colleagues in a recent review article (Desmedt et al., 2023). These studies demonstrate modality-specific routes of neural sensing, further substantiating the behavioral dissociations observed in interoceptive tasks.

### 2.3 Differential associations with affective and clinical variables

Another line of evidence for the dissociability of interoceptive modalities comes from their differential associations with affective and clinical phenomena. If interoception were a unitary empirical phenomenon, one would expect similar patterns of associations across domains. However, this is often not the case. For example, while some studies have found heightened cardiac interoceptive accuracy in anxiety disorders (Ehlers and Breuer, 1992; Pollatos et al., 2005), others have reported no differences or even reduced accuracy (Asmundson et al., 1993; De Pascalis et al., 2021). Thermoceptive accuracy was found to be increased in individuals with higher anxiety (Crucianelli et al., 2024). In contrast, respiratory interoception appears to be more consistently impaired in anxiety (Paulus and Stein, 2010; De Peuter et al., 2004). These discrepancies suggest that the role of interoception in anxiety is most likely modality-dependent. Similarly, depression has been linked to reduced interoceptive accuracy in some domains, such as gastric perception (Park et al., 2022; Avery et al., 2015) and thermoception (Terhaar et al., 2010; Crucianelli et al., 2024), but not others, such as cardioception (Dunn et al., 2010). Eating disorders also show divergent patterns, with some studies reporting decreased interoceptive accuracy (Pollatos et al., 2008) and others reporting varying relationships (Klabunde et al., 2013; Khalsa et al., 2015).

The differential associations between interoceptive modalities and clinical symptoms suggest that psychopathology does not involve a global interoceptive deficit, but rather selective impairments in specific channels. Table 1 highlights some of these incongruent relationships between affective variables and accuracy of different modalities of interoception. Importantly, associations between objective interoceptive accuracy and subjective symptom reports are often dissociated (De Pascalis et al., 2021; Paulus and Stein, 2010). Individuals with high anxiety may report heightened interoceptive sensations even if their accuracy is unimpaired or reduced (Petersen et al., 2015). Interestingly, this discrepancy between self-perceived and objectively measured interoceptive ability is not limited to anxiety disorders. Studies have shown that experienced meditators, despite their belief in enhanced interoceptive abilities, often perform no better than controls on objective measures of interoception (Khalsa et al., 2008). This highlights the importance of distinguishing between objective and subjective facets of interoception, which may have distinct clinical correlates (Heim et al., 2023).

**Table 1:** Relationship between affective and clinical variables and interoceptive accuracy across modalities.

**Interoceptive modality**	**Anxiety**	**Depression**	**Emotional arousal**	**Eating disorders**
Cardioception (heartbeat perception task)	Variable change (Adams et al., 2022; Kandiah et al., 2022)	Variable change (Dunn et al., 2010)	Accuracy ↑ (Pollatos et al., 2005; Wiens et al., 2000)	Variable accuracy (Khalsa et al., 2015; Pollatos et al., 2008)
Respiroception (occlusion counting task)	Accuracy ↓ (Garfinkel et al., 2016)	Accuracy ↓ (Chan et al., 2023)	Variable accuracy (Chan et al., 2023)	Variable accuracy (Khalsa et al., 2015)
Thermoception (dynamic thermal matching task)	Accuracy ↑ (Crucianelli et al., 2024)	Accuracy ↓ (Terhaar et al., 2010; Crucianelli et al., 2024)	Accuracy ↑ (Crucianelli et al., 2024)	–
Gastric perception (water load test)	–	Accuracy ↓ (Park et al., 2022)	Accuracy ↑ (Critchley and Garfinkel, 2017)	Accuracy ↓ (Klabunde et al., 2017)

This table highlights selected examples to illustrate disparities in the relationship between affective variables and interoceptive accuracy in differing modalities, rather than providing comprehensive review of the landscape. The ↑ and ↓ symbols respectively mean increase or decrease in interoceptive accuracy.

### 2.4 Lack of transfer of interoceptive training

If interoception existed as a unitary ability, one would expect training in one modality to transfer to others. However, evidence for such transfer is limited. Interoceptive interventions typically show domain-specific effects without generalizing to other modalities. For instance, cardiac biofeedback improves heartbeat perception (Goessl et al., 2017) without enhancing respiratory or gastrointestinal awareness.

Meditation practices, while offering a unique perspective on interoception, further illustrate this lack of transfer. Despite being associated with increased insular gray matter (Hölzel et al., 2011), their impact on interoceptive accuracy varies across traditions and modalities, highlighting the specificity of interoceptive training. Mindfulness-based interventions emphasizing breath awareness increase respiratory interoception (Daubenmier et al., 2013) but show inconsistent effects on cardiac perception (Parkin et al., 2014), underscoring the modality-specific nature of these improvements.

The study by Khalsa et al. (2008) provides additional evidence for this lack of transfer. Tibetan Buddhist and Kundalini practitioners showed no improvement in heartbeat detection tasks despite increased confidence in their abilities. While these practices may incorporate some bodily awareness, they don't explicitly emphasize it, and when present, it tends to focus more on breath than heartbeat. This suggests that even within meditation practices, interoceptive improvements may be limited to the specific modalities emphasized in training.

It's worth noting that the full extent of interoceptive enhancements in meditators may not yet be fully captured by current research methodologies. Meditators often report heightened sensitivity to subtle internal sensations (Mylius et al., 2023) and experiences of diminished body boundaries and altered time perception during meditation (Linares Gutiérrez et al., 2022; Thönes and Wittmann, 2016). These subjective experiences, while intriguing, do not necessarily indicate a transfer of interoceptive skills across different physiological systems.

The consistent lack of transfer across interoceptive modalities suggests distinct neural and cognitive mechanisms underlying different aspects of interoception. This emphasizes the need for modality-specific interventions in clinical applications. For example, respiratory biofeedback may benefit anxiety disorders with dysfunctional breathing patterns (Meuret et al., 2005), while gastrointestinal-focused interventions could be more relevant for eating disorders (Khalsa et al., 2022). These targeted approaches acknowledge the specificity of interoceptive training and its limited transfer across modalities.

## 3 Implications and future directions

The evidence reviewed above challenges the notion of interoception as a unitary construct and highlights the dissociability of interoceptive abilities across modalities. This has important implications for the conceptualization, measurement, and clinical application of interoception.

1. First, researchers should be cautious about generalizing findings from one interoceptive domain to others. Deficits or enhancements in a specific modality may not imply similar patterns in other channels. Ideally, studies should systematically assess multiple interoceptive modalities to capture a more comprehensive profile of an individual's interoceptive abilities.
2. Second, the development of interoceptive measures should focus on modality-specific tasks with established validity and reliability. The use of single tasks as proxies for global interoceptive ability is problematic given the lack of cross-modal convergence. Multidimensional batteries tapping into different facets of interoception within each modality (e.g., accuracy, sensitivity, awareness) may provide a more accurate assessment.
3. Third, clinical models of interoception should move beyond a one-size-fits-all approach and consider the specific interoceptive profiles associated with different disorders. Identifying the modalities that are selectively impaired or heightened in a given condition can inform more targeted interventions. For example, patients with generalized anxiety disorder may benefit from respiratory-focused training, while those with anorexia nervosa may require interventions targeting gastrointestinal perception.
4. Fourth, future research should investigate the mechanisms underlying the dissociability of interoceptive modalities. This may involve examining the neural substrates specific to each modality, the cognitive processes involved in different interoceptive tasks, and the factors that influence individual differences in interoceptive abilities. Longitudinal studies tracking the development of interoceptive abilities across modalities could also shed light on their divergence.
5. Fifth, researchers should consider the complex relationship between subjective interoceptive experiences and objective measures of interoceptive accuracy. For instance, studies on meditators have shown that their somatosensory perception is more sensitive to subtle internal sensations, potentially leading to both enhanced detection of bodily signals and misinterpretation of these signals (Mylius et al., 2023). Future research should aim to disentangle the effects of attention, expectation, and actual physiological sensitivity in different populations and across various interoceptive modalities.

While we argue against the notion of a unitary interoceptive ability, we do not discount the value of interoception as a heuristic construct. Furthermore, while the evidence showcased herein primarily examines accuracy across interoceptive modalities, we acknowledge that interoception encompasses multiple dimensions beyond accuracy (e.g., awareness, sensibility, attention, intensity). Even within a single modality, these dimensions can dissociate—as demonstrated by meditators showing heightened interoceptive awareness despite no improvement in cardiac accuracy (Khalsa et al., 2008). Our argument for the non-unitary nature of interoception, while grounded in accuracy measures, opens broader questions about potential dissociations across other dimensions of interoceptive experience. Interoception remains a useful umbrella term for organizing research on inner body perception and its role in cognition, affect, and behavior. However, researchers should be clear about the level of analysis they are operating at and not conflate modality-specific findings with general claims about interoception.

## Footnotes

^1^It is important to note that the inclusion of thermal signals within the domain of interoception is not universally accepted (Craig, 2002; Chen et al., 2021; Crucianelli et al., 2021, 2024). Some researchers argue that thermoception should be considered exteroceptive, as thermal receptors can be activated by external temperature changes. We acknowledge this debate and include thermal signals in our discussion to highlight the complexities and nuances in defining interoception.

^2^The Respiratory Occlusion Detection Task involves mild, brief airflow disruptions to assess respiratory interoceptive sensitivity, while gastric balloon distension uses controlled stomach inflation to measure gastrointestinal interoception.
